# Beyond the home: how pet ownership drives stray animal rescue willingness through empathy and moral obligation

**DOI:** 10.3389/fvets.2026.1732246

**Published:** 2026-02-19

**Authors:** Huanhua Lu, Zaina Jianaer, Ruihan Chen, Yiqing Lu

**Affiliations:** 1 School of Marxism, China University of Geosciences (Beijing), Beijing, China; 2 Foreign Environmental Cooperation Centre, Ministry of Ecology and Environment, Beijing, China

**Keywords:** animal empathy, animal welfare, cognitive appraisal, human-animal interaction, moral obligation, pet ownership experience, stray animal rescue, structural equation modeling

## Abstract

**Introduction:**

The global stray animal crisis poses significant challenges to animal welfare, public health, and community harmony. Understanding the public's willingness to rescue stray animals is therefore critical for developing effective interventions. Integrating insights from veterinary humanities and social sciences, this study examines how the human-companion animal bond, operationalized as pet ownership experience, influences rescue willingness through a sequential psychological process.

**Methods:**

Data from 447 Chinese participants were collected via questionnaire and analyzed using structural equation modeling. The study examined the relationships among pet ownership experience, cognitive appraisals (perceived cuteness, suffering, and health risk), emotional empathy, moral obligation, and rescue willingness.

**Results:**

Pet ownership experience significantly increased rescue willingness by enhancing perceptions of stray animals' cuteness and suffering, which in turn strengthened animal empathy and moral obligation. A dual-path effect emerged for perceived health risk: while it generally reduced rescue willingness by weakening empathy and obligation, it also had a positive direct effect, suggesting that for some individuals, higher risk perception may signal situational urgency and motivate action.

**Discussion:**

These findings underscore that the bond formed with companion pets can be a powerful catalyst for broader animal welfare engagement. For veterinary professionals and animal welfare organizations, this study suggests that outreach strategies should leverage positive cognitive appraisals, empathetic responses, and a sense of moral responsibility, while simultaneously addressing health concerns through public education on safe rescue practices.

## Introduction

Stray animals, including abandoned pets and their offspring, represent a widespread global challenge with profound implications for animal welfare, veterinary public health, and ecological balance. Managing this crisis requires not only effective veterinary and shelter services but also a profound understanding of the factors that motivate the public to engage in proactive rescue behaviors. From the perspective of veterinary humanities and social sciences, public willingness to rescue stray animals is a crucial component in building harmonious and healthy human–animal communities. Therefore, systematically examining the psychological determinants of rescue behavior is essential for promoting public engagement and informing evidence-based animal welfare policies and outreach programs.

Whether individuals take proactive rescue actions toward stray animals is a complex decision-making process, influenced by a combination of demographic characteristics, cognitive appraisals, emotional responses, and moral motivations. Previous studies have identified gender and pet ownership experience as key demographic factors, with women and pet owners generally showing greater willingness to help animals (1–6). Furthermore, animal empathy has also been established as a critical emotional driver of helping behavior (1, 7–9). However, existing research often examines these factors in isolation, lacking an integrated framework that explains how they interconnect. Crucially, there is a significant gap in understanding how the human-companion animal bond—operationalized through pet ownership experience—serves as a foundational psychological resource that initiates a cascading cognitive-emotional-moral process, ultimately leading to prosocial behavior toward stray animals.

Drawing upon cognitive appraisal theory (10), the empathy–altruism hypothesis (11), and the value–belief–norm (VBN) theory (12), this study proposes a comprehensive multilayered psychological model to explain the formation of rescue willingness. We conceptualize this process as involving four distinct but interrelated levels: (1) demographic characteristics (pet ownership experience and gender), (2) cognitive appraisals (perceived animal cuteness, perceived animal suffering, and perceived health risk), (3) an emotional response (animal empathy), and (5) a moral motivational factor (moral obligation). While gender is included as a well-established correlate for comparative purposes, this study specifically emphasizes the role of pet ownership experience as a proxy for a positive human-animal relationship. We conceptualize that pet ownership experience enhances cognitive appraisals of stray animals (perceived cuteness and suffering), which in turn elicit emotional responses (animal empathy) and activate moral motivations (moral obligation), ultimately increasing rescue willingness.

To test this integrative model, we employ structural equation modeling (SEM), aiming to systematically reveal the psychological determinants of the public's willingness to rescue stray animals, with a particular focus on the pivotal role of the pet ownership experience. The findings will provide a theoretical foundation for veterinary professionals, animal rescue organizations, and policymakers to design targeted interventions that leverage positive human-animal relationships to promote stray animal welfare and community health.

## Theoretical basis and research hypotheses

### The foundational role of pet ownership experience and gender

Based on the contact hypothesis (13) and attachment theory (14, 15), positive interactions with pets can effectively reduce prejudice, increase intimacy, and foster positive attitudes. Individuals with pet ownership experience are more likely to regard animals as “family members”, and such intimate relationships generalize to greater compassion and care for animals in general. Empirical evidence indicates that compared with non-pet owners, pet owners display stronger sympathy and greater willingness to help animals (3–6). We argue that pet ownership experience is not merely a demographic factor but a proxy for a meaningful relationship that cultivates a generalized ethic of care. This relationship enhances individuals' sensitivity to animals' needs and suffering, thereby motivating helping behaviors. Thus, we posit that pet ownership experience will predict greater willingness to rescue stray animals.

According to social role theory (16), societal expectations of gender roles shape different behavioral patterns. A large body of research consistently shows that women exhibit higher levels of generalized empathy and nurturing tendencies (1, 2, 17–20), which extend not only to interpersonal relationships but also to human-animal interactions. For instance, studies have demonstrated that, compared with men, women hold more positive attitudes toward animals, are more supportive of animal welfare policies, and report stronger intentions to protect animals (1, 2). This difference may stem from societal expectations that assign women more nurturing and caregiving roles, making them more inclined to express and enact rescue behaviors.

Based on these theories and evidence, we propose the following hypotheses:

H1: pet ownership experience and gender significantly influence rescue willingness.

H1a: individuals with pet ownership experience show significantly higher rescue willingness than those without such experience.

H1b: compared with men, women show significantly higher rescue willingness.

### The impact of cognitive appraisals on the rescue willingness

Cognitive appraisals play a crucial role in individual decision-making, particularly in complex situations such as rescuing stray animals. According to Lazarus's (10) cognitive appraisal theory, individuals' responses to external stimuli depend on their primary and secondary appraisals. In the context of rescuing stray animals, this process is reflected in three key dimensions of cognitive appraisals: perceived animal cuteness, perceived animal suffering and perceived health risk.

Perceived animal cuteness refers to individuals' subjective evaluations and psychological responses to infant-like morphological and behavioral traits in animals that can elicit affection and caregiving behaviors (21). Its theoretical foundation lies in Lorenz's (21) “Kindchenschema”, which suggests that infantile features (e.g., round face, large eyes, small nose) automatically trigger nurturing instincts and positive emotions. This effect applies not only to human infants but also to animals with similar features. Sherman et al. (22) found that viewing images of cute animals made individuals more careful in their behavior, which facilitates caring for vulnerable infants or small animals. Glocker et al. (23) further demonstrated that infantile features not only bias attention but also significantly increase willingness to help, even when such help requires time or financial costs. Borgi and Cirulli (24) confirmed that perceived cuteness in animals significantly predicts individuals' desire to care for them. Therefore, we hypothesize that perceptions of stray animals' cuteness positively influence individuals' willingness to rescue them.

Perceived animal suffering refers to individuals' cognitive judgments of animals' negative states (25, 26). According to the VBN (12), when people become aware of others' suffering and feel responsible to act, their personal moral norms are activated. Thus, when individuals cognitively recognize that animals are in pain and in need of help, they are more likely to develop the motivation to intervene. Previous research has shown that stronger perceptions of animal suffering predict stronger empathic responses and greater willingness to protect animals (25–27). Therefore, perceptions of stray animals' suffering can positively influence willingness to rescue them.

Perceived health risk refers to individuals' subjective evaluations and judgments of the likelihood and severity of potential threats to their own physical and mental health in a given context (28, 29). In this study, it specifically refers to individuals' subjective evaluations of the potential threats that stray animals may pose to human health. According to protection motivation theory [PMT; (28)], when facing potential threats, individuals engage in threat appraisal (severity and vulnerability) and coping appraisal (self-efficacy and response efficacy). If they perceive the threat as severe and themselves as incapable of coping, they experience fear and avoidance (28). In the context of stray animal rescue, individuals evaluate the risks of being attacked or contracting diseases (threat appraisal) and their ability to safely intervene (coping appraisal). If they perceive high health risk and low coping capacity, avoidance behaviors may occur, hindering rescue willingness. Prior studies have found that risk perceptions can suppress individuals' altruistic behaviors (29–31). Research in the animal domain also shows that perceptions of zoonotic disease risks reduce public support for wildlife conservation (32, 33). Thus, stronger health risk perceptions may decrease willingness to rescue stray animals.

Based on these theories and empirical evidence, we propose the following hypotheses: H2: cognitive appraisals (perceived animal cuteness/perceived animal suffering/perceived health risk) significantly influence rescue willingness.

### Emotional response: the mediating role of animal empathy

Animal empathy refers to humans' emotional resonance with animals' situations, including the perception of and affective responses to animal suffering (34, 35). Previous neuroscientific research implied that cognitive appraisals of animals (including perceived animal cuteness, perceived animal suffering and perceived health risk) may influence individuals' empathy for animals (36–40). For example, Kringelbach et al. (36) found that infant schema features activate the orbitofrontal cortex, a region closely related to emotional evaluation and decision-making, implying that perceptions of cuteness may enhance empathic responses through the brain's reward system; multiple fMRI studies have demonstrated that observing others in pain activates the anterior insula (AI) and anterior cingulate cortex (ACC), regions that overlap with the neural substrates of one's own pain experience (37–39), implying that perceptions of animal suffering may elicit animal empathy through the mirror neuron system; Adolphs (40) showed that fear and risk perception rely primarily on the amygdala, and hyperactivation of the amygdala can inhibit activation in empathy-related brain regions (e.g., prefrontal cortex), implying that perceived health risk may suppress animal empathy via amygdala hyperactivation.

On the other hand, according to the empathy–altruism hypothesis (11), empathy for others' distress can elicit altruistic behavior. This principle extends to the context of animal protection: when an individual experiences empathy for an animal, it can stimulate the willingness to protect the animal (1, 7–9). For instance, Smith et al. (7) found that enhancing empathy for animals increased people's intention to protect them. Similarly, Telle and Pfister (8) showed that empathy for orangutans promoted moral attitudes and donation behaviors toward their conservation.

Based on above theories and empirical evidence, we propose the following hypotheses: H3: animal empathy may mediate the relationship between cognitive appraisals (perceived animal cuteness/perceived animal suffering/perceived health risk) and rescue willingness.

### Motivational factor: the chain mediating effect of moral obligation

Moral obligation refers to individuals' strong sense of personal responsibility to take action (41). According to the Norm Activation Model (NAM; 40) and the VBN theory (12), the emergence of moral behavior requires three conditions: awareness of consequences, ascription of responsibility, and perceived ability to reduce the threat. When individuals recognize harmful consequences and feel both responsible and capable of changing these outcomes, their personal moral norms are activated, producing a strong sense of “I ought to act” (12, 41). In the context of rescuing stray animals, this process unfolds as follows: individuals first perceive animals' cuteness and suffering (cognitive appraisals), which elicits emotional resonance (animal empathy), thereby activating personal moral norms and generating a strong sense of moral obligation. Conversely, perceived health risk may trigger moral disengagement mechanisms (e.g., “I am not trained, and rescuing may do more harm than good”), thus weakening moral obligation.

On the other hand, under the theory of planned behavior (42), moral obligation is considered part of the attitudinal and normative components that directly drive behavioral intentions. When moral obligation is sufficiently strong, individuals will strive to overcome obstacles and find solutions (42). Extensive research has confirmed that moral obligation is one of the strongest proximal predictors of prosocial behaviors such as environmental protection and charitable giving (43–45). Moreover, studies have shown that moral obligation mediates the relationship between empathy and prosocial behaviors. For example, Hong et al. (46) found that among teachers, empathy enhanced moral obligation, which in turn promoted fairness behaviors. Similarly, Berenguer (47) found that inducing empathy toward nature enhanced moral reasoning and intentions for environmental protection. A recent work further supported this view, showing that empathy strengthens pro-environmental behavior through personal moral norms (48). Collectively, these findings indicate that moral obligation is a critical bridge that transforms empathy into prosocial behavior, and it may play a similar mediating role between animal empathy and pro-animal behavior.

Taken together, the preceding discussions of animal empathy and moral obligation underscore the importance of distinguishing their respective roles within the proposed framework. Although animal empathy and moral obligation are conceptually related, they represent distinct psychological processes that operate at different functional stages within the proposed framework. Animal empathy primarily reflects an affective response characterized by emotional resonance with animals' situations and suffering, capturing the extent to which individuals emotionally “feel with” animals (11, 35). In contrast, moral obligation represents a normative and motivational construct, referring to an internalized sense of personal responsibility and duty to act, expressed as a perceived moral imperative (“I ought to help”) (12, 41).

Importantly, existing theories suggest that empathy alone may be insufficient to motivate sustained or effortful prosocial action unless it is transformed into a sense of moral responsibility (12, 41). Within the Norm Activation Model and the Value–Belief–Norm framework, empathic emotional arousal functions as a catalyst that activates personal moral norms, which in turn generate moral obligation and guide behavioral intentions (12, 41). Distinguishing animal empathy from moral obligation therefore allows the model to capture the full psychological sequence from emotional arousal to moral motivation, clarifying why multiple elements are theoretically necessary rather than redundant. Retaining both constructs enables a more precise representation of how cognitive appraisals are translated into rescue willingness through sequential emotional and moral processes (43, 47).

Based on these theories and empirical evidence, this study proposes that in rescue contexts, cognitive appraisals of animals' cuteness and suffering evoke empathic responses, which in turn activate moral obligation, ultimately fostering rescue willingness. Conversely, perceived health risk may weaken rescue willingness by suppressing empathy and moral obligation. This sequential chain illustrates the complete psychological process from cognitive appraisal to emotional response, to moral motivation, and finally to behavioral intention.

H4: cognitive appraisal (perceived animal cuteness/perceived animal suffering/perceived health risk) → emotional response → moral motivation → behavioral intention.

### The present study

**Figure 1:**
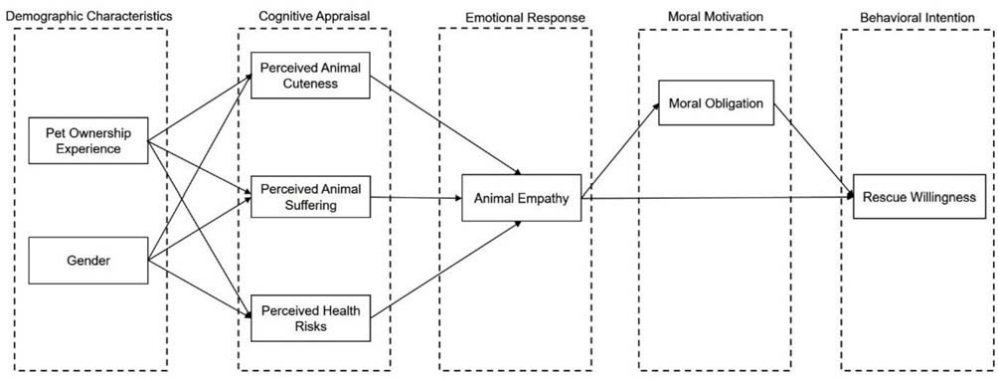
The theoretical model of the study. For clarity and visual simplicity, only the paths representing the hypothesized sequential mediation effects are illustrated. Not all direct and indirect effects between variables are depicted.

The present study integrated demographic characteristics (pet ownership experience and gender), cognitive appraisals, emotional responses, and moral obligation to construct a multilayered model (Figure 1) from pet ownership experience and gender to behavioral intentions. We hypothesized that pet ownership experience and gender would influence cognitive appraisals (perceived cuteness, perceived animal suffering, and perceived health risk), which in turn would elicit emotional responses (animal empathy) and moral obligation, ultimately shaping individuals' willingness to rescue stray animals (Figure 1). We conducted a survey to collect data and employed SEM to test the theoretical model (Figure 1). By revealing the interactions among demographic characteristics (pet ownership experience and gender), cognitive factors, emotional responses, and moral motivations, this study aims to deepen our understanding of human-animal interactions and also provide a theoretical foundation for designing more effective animal welfare advocacy strategies.

## Methods

### Participants

This study randomly selected one undergraduate university and one vocational college in China, and adopted convenience cluster sampling in compulsory public courses to collect data. Students who completed the survey received course credits as a reward. In addition, a snowball sampling technique was employed, whereby participants were invited to ask peers from other universities to participate. Data were collected via the Wenjuanxing online survey platform, yielding 460 responses. After excluding invalid responses (e.g., patterned answering, abnormally short completion times), 447 valid samples remained. Among them, 156 were male and 291 were female; 323 participants had previous pet ownership experience, and 124 did not. The mean age was 21.90 years (*SD* = 4.80). The study was approved by China University of Geosciences (Beijing) Psychological Ethics Committee (No. 20250305). All participants provided written informed consent before participating in this study.

### Measures

#### Perceived animal cuteness

Perceived animal cuteness was measured using the modified stray animal cuteness perception scale which measures the individual's perception of the cuteness, healing power and attractiveness of stray animals. The scale was modified from animal cuteness subscale of the Cuteness (Kawaii) Responsiveness Scale [CR-15; (49)]. It includes 5 items (e.g., “When I saw those little stray animals, I felt that they were all extremely adorable.”) rated on a 5-point Likert scale. The total score of five items was calculated as the indicator of perceived animal cuteness. The higher the total score is, the greater the degree of the individual's perception of the cuteness of stray animals. Cronbach's α is 0.94.

#### Perceived animal suffering

The modified stray animal pain perception scale is used to assess an individual's perception of the miserable situation of stray animals (e.g., “When I see a stray animal, I feel that it looks very painful.”). The scale is modified from the items related to animal pain perception in the Animal Attitude Scale (50). It contains three items, each rated on a 5-point Likert scale. The higher the total score, the stronger the individual's perception of the pain of stray animals. Cronbach's α is 0.93.

#### Perceived health risk

The modified perceived health risk scale was used to measure the degree of individuals' concern about potential health risk associated with contacting or adopting stray animals (e.g., “I am worried that the animal may carry zoonotic diseases such as rabies, leptospirosis, and toxoplasmosis.”). This scale, adapted from the Perceived Health Risk Scale (51), consists of three items, each rated on a 5-point Likert scale. A higher total score indicates that individuals perceive a higher level of health risk potentially posed by stray animals. Cronbach's α is 0.93.

#### Stray animal rescue willingness

A self-developed stray animal rescue willingness scale was used to measure participants' willingness to rescue stray animals. The six items assessed willingness to engage in behaviors such as sharing rescue information on social media, contacting animal rescue organizations, providing food/water/temporary shelter, donating money or supplies, paying for surgery for stray animals, and adopting stray animals. Items were rated on a 5-point Likert scale, with higher total scores indicating greater willingness to rescue stray animals. Cronbach's α was 0.92.

#### Animal empathy

The animal empathy scale was modified from the short version of the Empathic Concern Index (53, 54) to measure empathic responses toward stray animals. The three items assessed feelings of sympathy, concern, and compassion toward stray animals (e.g., “The plight of stray animals often makes me feel heartache and compassion.”), rated on a 5-point Likert scale. Higher total scores indicate greater empathy for stray animals. Cronbach's α was 0.96.

#### Moral obligation

In this study, the moral obligation items developed by Berenguer (47) based on the NAM (41) were modified and applied to the context of stray animal rescue. This modified scale assesses the degree to which individuals feel morally obligated to help stray animals (e.g., “Even if people around me do not support it, I still feel that I have a responsibility to help stray animals.”). The scale consists of five items, scored on a 5-point Likert scale. A higher total score indicates a stronger moral obligation to rescue stray animals. Cronbach's α is 0.94.

#### Demographic information

Participants reported demographic information including gender, age, education background, socioeconomic status, and pet ownership experience [“Have you ever owned a pet (currently or in the past)?”].

### Statistical analysis

First, descriptive statistics and correlation analyses were conducted using SPSS 25.0 to examine the associations among primary variables. Subsequently, SEM was performed in Mplus 8 to evaluate the hypothesized theoretical model. The significance of mediation effects was tested using a bootstrap approach with 5,000 resamples.

## Results

### Descriptive statistics

Descriptive statistics for the main variables are presented in Table 1. The absolute values of skewness and kurtosis for all variables are all below 1, which satisfies the prerequisite for parametric tests. Demographic variables (age, education, socioeconomic status) were unrelated to rescue willingness and were therefore not included in the subsequent model analyses.

**Table 1:** Descriptive statistical analysis.

**Variable**	***M***	***SD***	***Skewness***	***Kurtosis***
Perceived animal cuteness	16.78	5.28	−0.286	−0.324
Perceived animal suffering	10.83	3.02	−0.645	0.297
Perceived health risk	7.17	3.04	0.442	−0.045
Animal empathy	11.31	3.02	−0.705	0.485
Moral obligation	17.63	4.74	−0.462	0.639
Rescue willingness	20.55	5.38	−0.512	0.322

### Common method bias test

Harman's single-factor test indicated that the first principal component accounted for 33.70% of the variance, below the 40% threshold (52), suggesting no serious common method bias.

### Effects of pet ownership experience and gender

**Figure 2:**
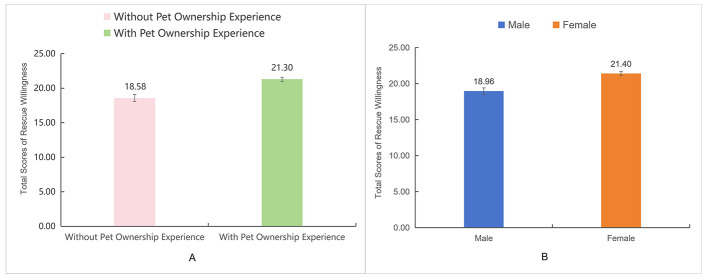
**(A, B)** Pet ownership experience difference and gender difference on rescue willingness.

Independent-samples *t*-tests revealed that participants with pet ownership experience (*M* = 21.30, *SD* = 5.04) showed higher rescue willingness than those without pet ownership experience (*M* = 18.58, *SD* = 5.74), *t*(447) = −4.91, *p* < 0.001, Cohen's *d* = 0.52 (Figure 2A); females (*M* = 21.40, *SD* = 5.08) reported significantly higher rescue willingness than males (*M* = 18.96, *SD* = 5.58), *t*(447) = −4.69, *p* < 0.001, Cohen's *d* = 0.46 (Figure 2B).

### Correlation analysis

Pet ownership experience was significantly correlated with rescue willingness (*r* = 0.227, *p* < 0.001, Table 2), perceived animal cuteness (*r* = 0.223, *p* < 0.001, Table 2), perceived animal suffering (*r* = 0.218, *p* < 0.001, Table 2), animal empathy (*r* = 0.184, *p* < 0.001, Table 2), and moral obligation (*r* = 0.167, *p* < 0.001, Table 2), but not with perceived health risk (*r* = −0.080, *p* = 0.093, Table 2).

**Table 2:** Correlation analysis.

	**Pet ownership experience**	**Perceived animal cuteness**	**Perceived animal suffering**	**Perceived health risk**	**Animal empathy**	**Moral obligation**	**Rescue willingness**	**Gender**
Pet ownership experience	—							
Perceived animal cuteness	0.223^***^	—						
Perceived animal suffering	0.218^***^	0.623^***^	—					
Perceived health risk	−0.080	−0.241^***^	−0.395^***^	—				
Animal empathy	0.184^***^	0.620^***^	0.633^***^	−0.443^***^	—			
Moral obligation	0.167^***^	0.659^***^	0.588^***^	−0.456^***^	0.821^***^	—		
Rescue willingness	0.227^***^	0.709^***^	0.620^***^	−0.249^***^	0.762^***^	0.745^***^	—	
Gender	0.112^*^	0.117^*^	0.069	0.107^*^	0.106^*^	0.124^**^	0.217^***^	—

^*^p < 0.05, ^**^p < 0.01, ^***^p < 0.001.

Gender was positively correlated with perceived animal cuteness (*r* = 0.117, *p* = 0.014, Table 2), perceived health risk (*r* = 0.107, *p* = 0.024, Table 2), animal empathy (*r* = 0.106, *p* = 0.026, Table 2), moral obligation (*r* = 0.124, *p* = 0.009, Table 2), and rescue willingness (*r* = 0.217, *p* < 0.001, Table 2), but not with perceived animal suffering (*r* = 0.069, *p* = 0.147, Table 2).

Perceived animal cuteness (*r* = 0.709, *p* < 0.001, Table 2) and perceived animal suffering (*r* = 0.620, *p* < 0.001, Table 2) were positively correlated with rescue willingness, while perceived health risk was negatively correlated (*r* = −0.249, *p* < 0.001, Table 2). Animal empathy (*r* = 0.762, *p* < 0.001, Table 2) and moral obligation (*r* = 0.745, *p* < 0.001, Table 2) were also positively correlated with rescue willingness.

### Structural equation model results

#### Model fit

SEM analysis was constructed by Mplus 8.0 software to test the theoretical model proposed in this study (Figure 1). Although the theoretical model (Figure 1) illustrates only the hypothesized sequential mediation paths for clarity, the statistical analyses were conducted using a full model (*M*_0_) that included all possible direct effects and indirect effects between the variables. The full model (*M*_0_) exhibited acceptable fit, with some indices reaching good levels and others falling within acceptable ranges (χ^2^/df = 4.588, CFI = 0.994, TLI = 0.946, RMSEA = 0.090, SRMR = 0.027, Table 3).

**Table 3:** Fit indices for the hypothesized model and the simplified model.

**Model**	**Description**	**χ^2^/df**	**CFI**	**TLI**	**RMSEA**	**SRMR**
*M*_0_	Full model (all paths)	4.588	0.994	0.946	0.090	0.027
*M*_1_	Final simplified model	2.747	0.993	0.974	0.063	0.030

*M*_0_, model specifying all hypothesized paths; *M*_1_, Final model after removing some non-significant paths from *M*_0_.

To enhance model parsimony, some non-significant paths were removed, resulting in a simplified model (*M*_1_). The results show that all fit indices of the simplified model (*M*_1_) are satisfactory (χ^2^/df = 2.747, CFI = 0.993, TLI = 0.974, RMSEA = 0.063, SRMR = 0.030), indicating that it has better and more parsimonious explanatory power for the data. Accordingly, the simplified model (*M*_1_) was retained as the final model for substantive interpretation.

#### Path coefficients

**Figure 3:**
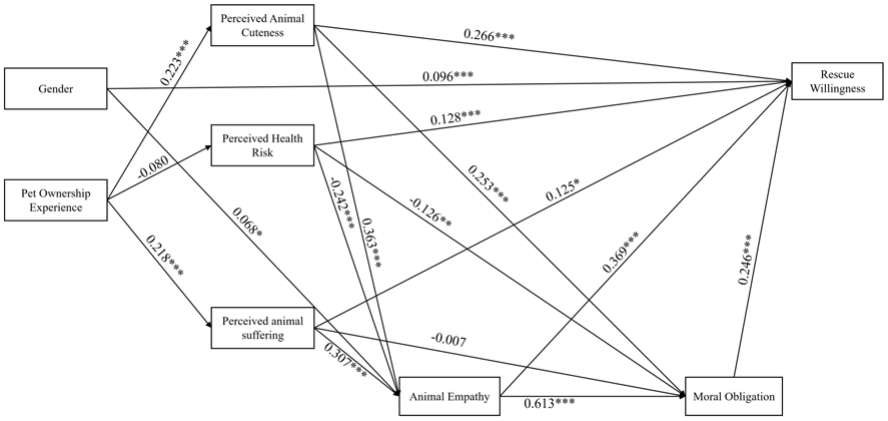
Standardized path coefficients for the Final Model (M_1_). The results of the SEM analysis displaying standardized path coefficients. The value indicated on the paths are the standardized estimates (β). ^***^p < 0.001 ^**^p < 0.01, ^*^p < 0.05.

The simplified model (*M*_1_) was used to test the relationships among the variables, so as to verify our research hypotheses. The standardized path coefficients are presented in Figure 3.

Pet ownership experience significantly predicted perceived animal cuteness (β = 0.223, *p* < 0.001, Figure 3) and perceived animal suffering (β = 0.218, *p* < 0.001, Figure 3), but did not significantly predict perceived health risk (β = −0.080, *p* = 0.121, Figure 3). Gender significantly predicted animal empathy (β = 0.068, *p* = 0.027, Figure 3) and rescue willingness (β = 0.096, *p* < 0.001, Figure 3).

In terms of cognitive appraisals, perceived animal cuteness significantly predicted animal empathy (β = 0.363, *p* < 0.001, Figure 3), moral obligation (β = 0.253, *p* < 0.001, Figure 3), and rescue willingness (β = 0.266, *p* < 0.001, Figure 3); perceived animal suffering was a significant positive predictor of animal empathy (β = 0.307, *p* < 0.001, Figure 3) and rescue willingness (β = 0.125, *p* = 0.014, Figure 3), but not moral obligation (β = −0.007, *p* = 0.878, Figure 3); perceived health risk negatively predicted animal empathy (β =-0.242, *p* < 0.001, Figure 3) and moral obligation (β = −0.126, *p* = 0.001, Figure 3), yet was a significant positive predictor of rescue willingness (β = 0.128, *p* < 0.001, Figure 3).

In addition, animal empathy (β = 0.369, *p* < 0.001, Figure 3) and moral obligation (β= 0.246, *p* < 0.001, Figure 3) both significantly positively predicted rescue willingness. Animal empathy also significantly positively predicted moral obligation (β = 0.613, *p* < 0.001, Figure 3).

#### Indirect effects

Bootstrap analysis (5,000 samples) confirmed significant indirect effects (see Table 4). Pet ownership experience indirectly increased rescue willingness through perceived animal cuteness [β = 0.059, 95% CI (0.032, 0.096), Table 4] and perceived animal suffering [β = 0.027, 95% CI (0.006, 0.060), Table 4], but not through perceived health risk [β = −0.010, 95% CI (−0.026, 0.002), Table 4]. Significant chain mediation pathways included: through perceived animal cuteness → animal empathy [β = 0.030, 95% CI (0.015, 0.052), Table 4], perceived animal cuteness → moral obligation [β = 0.014, 95% CI (0.006, 0.028), Table 4], perceived animal cuteness → animal empathy → moral obligation [β = 0.012, 95% CI (0.006, 0.024), Table 4], perceived animal suffering → animal empathy [β = 0.025, 95% CI (0.012, 0.045), Table 4], and perceived animal suffering → animal empathy → moral obligation [β = 0.010, 95% CI (0.004, 0.020), Table 4]. No paths through perceived health risk were significant. Overall, pet ownership experience increased perceived animal cuteness and suffering, which in turn enhanced animal empathy and moral obligation, thereby promoting willingness to rescue stray animals.

**Table 4:** Indirect effects for the final model (*M*_1_).

**Type of effect**	**Pathway**	**β**	**95%CI**	
			**Lower**	**Upper**
**Total indirect effect (POE** ** → RW)**		0.179	0.105	0.251
Simple mediation	POE → PAC → RW	0.059	0.032	0.096
	POE → PAS → RW	0.027	0.006	0.060
	POE → PHR → RW	−0.010	−0.026	0.002
Single-mediator chain	POE → PAC → AE → RW	0.030	0.015	0.052
	POE → PAS → AE → RW	0.025	0.012	0.045
	POE → PHR → AE → RW	0.007	−0.001	0.020
	POE → PAC → MO → RW	0.014	0.006	0.028
	POE → PAS → MO → RW	0.000	−0.006	0.005
	POE → PHR → MO → RW	0.002	−0.001	0.008
Dual-mediator chain	POE → PAC → AE → MO → RW	0.012	0.006	0.024
	POE → PAS → AE → MO → RW	0.010	0.004	0.020
	POE → PHR → AE → MO → RW	0.003	−0.001	0.009
**Total indirect effect (Gender** ** → RW)**		0.035	0.005	0.071
	Gender → AE → RW	0.025	0.003	0.052
	Gender → AE → MO → RW	0.010	0.002	0.024
**Total indirect effect (PAC → RW)**		0.250	0.173	0.334
	PAC → AE → RW	0.134	0.085	0.189
	PAC → MO → RW	0.062	0.029	0.110
	PAC → AE → MO → RW	0.055	0.028	0.093
**Total indirect effect (PAS** ** → RW)**		0.158	0.088	0.232
	PAS → AE → RW	0.113	0.067	0.170
	PAS → MO → RW	−0.002	−0.026	0.020
	PAS → AE → MO → RW	0.046	0.023	0.079
**Total indirect effect (PHR** ** → RW)**		−0.157	−0.228	−0.103
	PHR → AE → RW	−0.089	−0.143	−0.050
	PHR → MO → RW	−0.031	−0.060	−0.013
	PHR → AE → MO → RW	−0.036	−0.066	−0.018

POE, pet ownership experience; PAC, perceived animal cuteness; PHR, perceived health risk; PAS, perceived animal suffering; AE, animal empathy; MO, moral obligation; RW, rescue willingness.

Gender indirectly affected rescue willingness through animal empathy [β = 0.025, 95% CI (0.003, 0.052), Table 4] and through animal empathy → moral obligation [β = 0.010, 95% CI (0.002, 0.024), Table 4].

Perceived animal cuteness indirectly increased rescue willingness through animal empathy [β = 0.134, 95% CI (0.085, 0.189), Table 4], moral obligation [β = 0.062, 95% CI (0.029, 0.110), Table 4], and animal empathy → moral obligation [β = 0.055, 95% CI (0.028, 0.093), Table 4]. Perceived animal suffering increased rescue willingness through animal empathy [β = 0.113, 95% CI (0.067, 0.170), Table 4] and animal empathy → moral obligation [β = 0.046, 95% CI (0.023, 0.079), Table 4], but not through moral obligation alone. Perceived health risk reduced rescue willingness through animal empathy [β = −0.089, 95% CI (−0.143, −0.050), Table 4], moral obligation [β = −0.031, 95% CI (−0.060, −0.013), Table 4], and animal empathy → moral obligation [β = −0.036, 95% CI (−0.066, −0.018), Table 4].

These results suggested that pet ownership experience enhanced rescue willingness by increasing perceived animal cuteness and suffering, which subsequently boosted animal empathy and moral obligation. Perceived health risk did not mediate this relationship. Gender influenced rescue willingness indirectly through animal empathy and moral obligation. Cognitive appraisals also played key roles: perceived animal cuteness promoted rescue willingness through animal empathy and moral obligation; perceived animal suffering did so through animal empathy (but not moral obligation alone); and perceived health risk reduced rescue willingness by diminishing animal empathy and moral obligation.

## Discussion

This study aimed to elucidate the psychological mechanisms underlying the public's willingness to rescue stray animals through a multilayered model, with a specific focus on the role of the human-companion animal bond, as measured by pet ownership experience. The findings largely supported our hypothesized model, revealing a clear sequential pathway from pet ownership experience to cognitive appraisal, to emotional and moral engagement, and finally to behavioral intention. The findings provide valuable insights for the field of veterinary humanities and social sciences, highlighting the importance of integrating psychological understanding into animal welfare initiatives.

First, the primary finding of this study is that pet ownership experience, as a manifestation of the human-companion animal bond, serves as a key factor driving individuals' willingness to rescue stray animals. The significant total indirect effect of pet ownership experience underscores its powerful role in initiating the entire psychological process that leads to rescue willingness. This discovery greatly expands our understanding of contact hypothesis (13) and attachment theory (14, 15): positive interactions and emotional bonds with companion animals not only foster affection for specific animals but also generalize into a more widespread compassion, heightened sensitivity, and stronger sense of responsibility toward animals as a whole. Specifically, individuals with pet ownership experience reported higher perceptions of stray animals' cuteness and suffering. This indicates that the bond with a pet hones one's ability to recognize appealing and vulnerable traits in animals, making individuals more attuned to the needs of strays. The non-significant effect on perceived health risk is intriguing. This may be because individuals with pet ownership experience, while more knowledgeable about animals, are also more aware of potential risks (e.g., zoonotic diseases), and these opposing cognitions may cancel each other out, resulting in no significant difference from those without such experience. The findings extend the implications of the human-companion animal bond beyond the household context, suggesting that positive relationships with pets can foster a generalized ethic of care toward animals in need. For veterinary practices and animal welfare organizations, this underscores the potential of engaging pet owners as advocates and active participants in community-based stray animal management programs. These individuals may be more receptive to educational campaigns and volunteer opportunities due to their heightened sensitivity to animal suffering and cuteness.

More importantly, the results further elucidate the specific mechanisms through which the pet ownership experience operates. The sequential mediation paths—particularly through perceived cuteness/suffering → animal empathy → moral obligation—strongly support the integration of cognitive appraisal theory (10), the empathy-altruism hypothesis (11), and the NAM (41). It appears that pet ownership experience first shapes how one sees stray animals (cognitive appraisal), which triggers an emotional resonance (empathy), which in turn activates a deep sense of responsibility to act (moral obligation). This cascade effectively translates pet ownership experience into a motivational force for prosocial behavior.

A particularly intriguing finding concerns the complex dual-path mechanism of perceived health risk. On one hand, consistent with theoretical expectations, it exerted a significant negative indirect effect on rescue willingness by suppressing animal empathy and moral obligation, aligning with PMT (28), which posits that concerns for personal safety can serve as major psychological barriers to rescue behavior. On the other hand, after controlling the mediating paths, it exhibited a significant positive direct effect. This suggests that among individuals perceiving the same levels of cuteness and suffering, those perceiving higher health risk tended to show greater rescue willingness. One plausible explanation is that high risk may highlight the urgency and severity of the situation, thereby activating courage, motivating individuals to overcome fear and engage in rescue. This tension between “suppressing empathy” and “activating courage” reveals the psychological complexity of altruistic decision-making under risk. This complexity indicates that public health messaging must be nuanced. Rather than solely emphasizing risks, which might paralyze action, communications should pair risk awareness with clear, accessible guidelines on safe and humane handling of stray animals, provided in collaboration with veterinary experts. Empowering the public with knowledge and self-efficacy (e.g., through short demonstrations on safe approach techniques) can help mitigate fear and transform concern into constructive action.

A core finding is that animal empathy and moral obligation serve as key mediators. These results strongly support an integrated framework of the empathy-altruism hypothesis (11) and the NAM (41). Cognitive appraisal first elicits animal empathy, which then transforms into and activates a deeper, responsibility-based moral obligation. Ultimately, it is this moral motivation of “I feel responsible to act” that serves as the most direct and powerful proximal driver of behavioral intention. For perceived animal suffering, the effect must fully operate through empathy to effectively translate into moral obligation and action intention, highlighting the central role of emotional resonance in the moral activation process.

## Theoretical implications

First, by using structural equation modeling, we systematically elucidated the complete psychological chain of “demographic characteristics (pet ownership experience and gender) → cognitive appraisal → emotional response → moral motivation → behavioral intention”. This provides an integrated framework for understanding pro-animal behavior in human–animal interactions and addresses the fragmentation of prior research that typically examined single variables in isolation.

Second, this study revealed the dual-path effect of perceived health risk on rescue willingness: it negatively predicted willingness by suppressing empathy and moral obligation, while potentially positively predicted willingness by highlighting situational urgency and activating courage. This complex mechanism offers a novel perspective for understanding pro-animal behavior in risk-laden contexts.

Finally, by situating the study in the Chinese cultural context, this research not only validates the applicability of Western theories in China but also highlights the critical role of cultural factors in shaping human rescue willingness. The findings contribute to cross-cultural comparisons and the theoretical development of human–animal interactions and animal welfare research.

## Practical implications

First, the results showed that enhancing public perception of animal cuteness and suffering can effectively stimulate empathy and rescue willingness. Animal welfare organizations should leverage the “baby schema” effect when designing publicity materials, presenting stray animals as cute and innocent, while emphasizing narratives that depict their suffering and distress, thereby eliciting empathic responses and moral obligation from the public.

Second, the inhibitory effect of perceived health risk on empathy and moral obligation indicates that veterinary and rescue organizations should develop and widely disseminate standardized educational resources on zoonotic disease prevention and safe rescue protocols. This can reduce the barrier posed by health risk perceptions.

Third, the positive effect of moral obligation suggests that publicity strategies emphasizing personal responsibility and social norms (e.g., “Your help is crucial to its survival”) may be more effective in promoting actual rescue behaviors than mere information dissemination.

Finally, the findings indicate that interventions should be tailored to specific groups. For individuals with pet ownership experience and females, efforts could focus on enhancing and reinforcing existing empathy and moral responsibility. For risk-sensitive groups, communication strategies should emphasize safety knowledge and address concerns effectively.

## Limitations and future directions

This study has several limitations. First, data were collected through self-report, which may be subject to social desirability bias. Future research could employ situational experiments or observational methods to assess actual rescue behavior. Second, the cross-sectional design limits causal inferences. Future research could adopt longitudinal designs or experimental approaches to further validate causal relationships among the variables. Third, although several demographic variables were measured, the present study did not adopt an intersectional analytic approach. Preliminary analyses indicated that age, education, and socioeconomic status were not significantly associated with rescue willingness and were therefore not included in the subsequent model analyses. While this analytic decision was theoretically and statistically justified for the purposes of model parsimony, it may limit the extent to which nuanced interactions among demographic characteristics can be captured. Future research could employ intersectional or stratified designs, recruit more demographically diverse samples, or apply multi-group and interaction-based analytic approaches to examine how demographic characteristics jointly influence rescue-related cognitions, emotions, and moral motivations. Fourth, the sample was characterized by a higher proportion of female participants and a relatively young mean age (21.9 years), which reflects the demographic composition of the recruited population. Although gender was retained as a focal predictor in the model and age was not significantly associated with rescue willingness in preliminary analyses, this sample composition may limit the generalizability of the findings to broader or more demographically balanced populations. Future research could benefit from recruiting more age-diverse samples and adopting more gender-balanced designs to further examine the robustness of the proposed psychological mechanisms across demographic groups.

Finally, this study primarily focused on individual psychological factors. Future research could incorporate additional situational factors (e.g., time pressure, social norms and the availability of local veterinary support services) to provide a more comprehensive understanding of rescue willingness within a community ecology framework.

## Conclusion

In conclusion, this study revealed the multi-level psychological mechanisms underlying public willingness to rescue stray animals, highlighting the foundational role of the pet ownership experience and the central roles of cognitive appraisal, animal empathy, and moral obligation. Pet ownership experience serves as a critical catalyst, enhancing perceptions of stray animals' cuteness and suffering, which in turn spark empathy and a sense of moral obligation, ultimately motivating rescue willingness. For those working in veterinary medicine, animal welfare, and public health, these findings highlight the importance of designing interventions that thoughtfully engage the public's cognitive, emotional, and moral faculties. By doing so, we can more effectively harness community compassion to improve the welfare of stray animals and foster healthier human-animal ecosystems.

## Data Availability

The datasets presented in this study can be found in online repositories. The names of the repository/repositories and accession number(s) can be found below: data will be made available on https://osf.io/ydv4u?view_only=003a4abb019449e8a012f8de8449f663.

## References

[1] Taylor N Signal TD. Empathy and attitudes to animals. Anthrozoös. (2005) 18:18–27. doi: 10.2752/089279305785594342

[2] Phillips C Izmirli S Aldavood SJ Alonso M Choe BI Hanlon A . An international comparison of female and male students' attitudes to the use of animals. Animals. (2011) 1:7–26. doi: 10.3390/ani1010007 26486211 PMC4552214

[3] Liang Y Meng C Chen R Yang Y Zeng Y. Pet ownership and its influence on animal welfare attitudes and consumption intentions among Chinese university students. Animals. (2024) 14:3242. doi: 10.3390/ani14223242 39595295 PMC11591475

[4] Frampton GA Oliva JL. Support for the ‘Pets as Ambassadors' hypothesis in men: higher animal empathy in Australian pet-ownersvs non-owners and farmers. Anim Welf. (2024) 33:e23. doi: 10.1017/awf.2024.25 38721623 PMC11076914

[5] Barklam EB Felisberti FM. The influence of pet owners' empathy on perceptions of dog and cat distress vocalizations and caregiving behaviors. J Appl Anim Welf Sci. (2024) 29:1–15. doi: 10.1080/10888705.2024.2427673 39562511

[6] Paul ES Serpell JA. Childhood pet keeping and humane attitudes in young adulthood. Anim Welf. (1993) 2:321–37. doi: 10.1017/S0962728600016109

[7] Smith P Mann J Marsh A. Empathy for wildlife: the importance of the individual. Ambio. (2024) 53:1269–80. doi: 10.1007/s13280-024-02017-4 38795282 PMC11300747

[8] Telle NT Pfister HR. Empathy and donation behavior toward happy and sad chimpanzees. Hum Anim Interact Bull. (2014) 2:1–20. doi: 10.1079/hai.2014.0012

[9] Gu X Xie L Bexell SM. The link between attitudes toward animals and empathy with humans in China: mediation of empathy with animals. Anthrozoös. (2023) 37:75–88. doi: 10.1080/08927936.2023.2266924

[10] Lazarus RS. Emotion and Adaptation. Oxford: Oxford University Press (1991). doi: 10.1093/oso/9780195069945.001.0001

[11] Batson CD. The Altruism Question: Toward a Social-Psychological Answer. Mahwah, NJ: Lawrence Erlbaum Associates, Inc. (1991).

[12] Stern PC Dietz T Abel T Guagnano GA Kalof L. A value-belief-norm theory of support for social movements: the case of environmentalism. Hum Ecol Rev. (1999) 6:81–97.

[13] Allport GW. The Nature of Prejudice. Addison-Wesley (1954).

[14] Bowlby J. Attachment and Loss: Vol. 1. Attachment. New York, NY: Basic Books (1969).

[15] Zilcha-Mano S Mikulincer M Shaver PR. Pet in the therapy room: an attachment perspective on animal-assisted therapy. Attach Hum Dev. (2011) 13:541–61. doi: 10.1080/14616734.2011.608987 22011099

[16] Eagly AH Wood W. Social role theory of sex differences. In:Naples NA, editors. The Wiley Blackwell Encyclopedia of Gender and Sexuality Studies. Hoboken, NJ: Wiley (2016). p. 1–3. doi: 10.1002/9781118663219.wbegss183

[17] Christov-Moore L Simpson EA Coudé G Grigaityte K Iacoboni M Ferrari PF. Empathy: gender effects in brain and behavior. Neurosci Biobehav Rev. (2014) 46:604–27. doi: 10.1016/j.neubiorev.2014.09.001 25236781 PMC5110041

[18] Prato-Previde E Basso Ricci E Colombo ES. The complexity of the human-animal bond: empathy, attachment and anthropomorphism in human-animal relationships and animal hoarding. Animals. (2022) 12:2835. doi: 10.3390/ani12202835 36290219 PMC9597799

[19] Herzog HA. Gender differences in human-animal interactions: a review. Anthrozoös. (2007) 20:7–21. doi: 10.2752/089279307780216687

[20] Gaspar A Esteves F. Empathy development from adolescence to adulthood and its consistency across targets. Front Psychol. (2022) 13:936053. doi: 10.3389/fpsyg.2022.936053 36300042 PMC9590310

[21] Lorenz K. Die angeborenen Formen möglicher Erfahrung [The innate forms of potential experience]. Z Tierpsychol. (1943) 5:235–409. German. doi: 10.1111/j.1439-0310.1943.tb00655.x

[22] Sherman GD Haidt J Coan JA. Viewing cute images increases behavioral carefulness. Emotion. (2009) 9:282–6. doi: 10.1037/a0014904 19348541

[23] Glocker ML Langleben DD Ruparel K Loughead JW Valdez JN Griffin MD . Baby schema modulates the brain reward system in nulliparous women. Proc Nat Acad Sci. (2009) 106:9115–9. doi: 10.1073/pnas.0811620106 19451625 PMC2690007

[24] Borgi M Cirulli F. Pet face: mechanisms underlying human–animal relationships. Front Psychol. (2016) 7:298. doi: 10.3389/fpsyg.2016.00298 27014120 PMC4782005

[25] Kielland C Skjerve E Østerås O Zanella AJ. Dairy farmer attitudes and empathy toward animals are associated with animal welfare indicators. J Dairy Sci. (2010) 93:2998–3006. doi: 10.3168/jds.2009-2899 20630216

[26] Luna D Vásquez RA Yáñez JM Tadich T. The relationship between working horse welfare state and their owners' empathy level and perception of equine pain. Anim Welf. (2018) 27:115–23. doi: 10.7120/09627286.27.2.115

[27] Bastian B Costello K Loughnan S Hodson G. When closing the human–animal divide expands moral concern: the importance of framing. Soc Psychol Personal Sci. (2012) 3:421–9. doi: 10.1177/1948550611425106

[28] Rogers RW. A protection motivation theory of fear appeals and attitude change. J Psychol. (1975) 91:93–114. doi: 10.1080/00223980.1975.9915803 28136248

[29] Slovic P. Perception of risk. Science. (1987) 236:280–5. doi: 10.1126/science.3563507 3563507

[30] Zeng Y Xiao G Ye B Zhang Y Liu M Wang X . The relationship between risk perception of COVID-19 and willingness to help: a moderated mediation model. Child Youth Serv Rev. (2022) 137:106493. doi: 10.1016/j.childyouth.2022.106493 35400776 PMC8983077

[31] Zhu Y Zhang S Ouyang M Zhang F Wang C. The moderating effect of COVID-19 risk perception on the relationship between empathy and volunteer behavior. Front Public Health. (2022) 10:863613. doi: 10.3389/fpubh.2022.863613 35784213 PMC9243539

[32] Decker DJ Siemer WF Wild MA Castle KT Wong D Leong KM Evensen DTN. Communicating about zoonotic disease: strategic considerations for wildlife professionals. Wildl Soc Bull. (2011) 35:112–19. doi: 10.1002/wsb.29

[33] Decker DJ Siemer WF Evensen DTN Stedman RC Mccomas KA Wild MA . Public perceptions of wildlife-associated disease: Risk communication matters. Hum Wildl Interact. (2012) 6:112–22. doi: 10.26077/8nz7-x725

[34] Mehrabian A Epstein N. A measure of emotional empathy. J Pers. (1972) 40:525–43. doi: 10.1111/j.1467-6494.1972.tb00078.x 4642390

[35] Paul ES. Empathy with animals and with humans: are they linked? Anthrozoös. (2000) 13:194–202. doi: 10.2752/089279300786999699

[36] Kringelbach ML Lehtonen A Squire S Harvey AG Craske MG Holliday IE . A specific and rapid neural signature for parental instinct. PLoS ONE. (2008) 3:e1664. doi: 10.1371/journal.pone.0001664 18301742 PMC2244707

[37] Brethel-Haurwitz KM Cardinale EM Vekaria KM Robertson EL Walitt B VanMeter JW . Extraordinary altruists exhibit enhanced self-other overlap in neural responses to distress. Psychol Sci. (2018) 29:1631–41. doi: 10.1177/0956797618779590 30130165 PMC6180668

[38] Gu X Gao Z Wang X Liu X Knight RT Hof PR . Anterior insular cortex is necessary for empathetic pain perception. Brain. (2012) 135:2726–35. doi: 10.1093/brain/aws199 22961548 PMC3437027

[39] Lamm C Decety J Singer T. Meta-analytic evidence for common and distinct neural networks associated with directly experienced pain and empathy for pain. Neuroimage. (2011) 54:2492–502. doi: 10.1016/j.neuroimage.2010.10.014 20946964

[40] Adolphs R. The biology of fear. Curr Biol. (2013) 23:R79–93. doi: 10.1016/j.cub.2012.11.055 23347946 PMC3595162

[41] Schwartz SH. Normative influences on altruism. In:Berkowitz L, editor, *Advances in Experimental Social Psychology*. Vol. 10. Cambridge, MA: Academic Press (1977). p. 221–79. doi: 10.1016/S0065-2601(08)60358-5

[42] Ajzen I. The theory of planned behavior. Organ Behav Hum Decis Process. (1991) 50:179–211. doi: 10.1016/0749-5978(91)90020-T

[43] Bamberg S Möser G. Twenty years after Hines, Hungerford, and Tomera: a new meta-analysis of psycho-social determinants of pro-environmental behaviour. J Environ Psychol. (2007) 27:14–25. doi: 10.1016/j.jenvp.2006.12.002

[44] Smith JR McSweeney A. Charitable giving: the effectiveness of a revised theory of planned behaviour model. J Commun Appl Soc Psychol. (2007) 17:363–85. doi: 10.1002/casp.906

[45] Harland P Staats H Wilke HAM. Explaining proenvironmental intention and behavior by means of the theory of planned behavior and personal norm. J Appl Soc Psychol. (1999) 29:2505–28. doi: 10.1111/j.1559-1816.1999.tb00123.x

[46] Hong Y Cai J Lan R Wang K Lian R Chen L. Empathy and teachers' fairness behavior: the mediating role of moral obligation and moderating role of social value orientation. PLoS ONE. (2022) 17:e0268681. doi: 10.1371/journal.pone.0268681 35679271 PMC9182229

[47] Berenguer J. The effect of empathy in proenvironmental attitudes and behaviors. Environ Behav. (2007) 39:269–83. doi: 10.1177/0013916506292937

[48] Ienna M Rofe A Gendi M Douglas HE Kelly M Hayward MW . The relative role of knowledge and empathy in predicting pro-environmental attitudes and behavior. Sustainability. (2022) 14:4622. doi: 10.3390/su14084622

[49] Takamatsu R. Measuring affective responses to cuteness and Japanese kawaii as a multidimensional construct. Curr Psychol. (2020) 39:1362–74. doi: 10.1007/s12144-018-9836-4

[50] Herzog H Grayson S McCord D. Brief measures of the animal attitude scale. Anthrozoös. (2015) 28:693–706. doi: 10.2752/089279315X14129350721894

[51] Cano S Chrea C Salzberger T Alfieri T Emilien G Mainy N . Perceived Risk Instrument–Perceived Health Risk Scale. Washington, DC: APA PsycTests (2018). doi: 10.1037/t70479-000 PMC6151038 30241527

[52] Podsakoff PM Organ DW. Self-reports in organizational research: problems and prospects. J Manage. (1986) 12:531–44. doi: 10.1177/014920638601200408

[53] Pfattheicher S Nielsen YA Thielmann I. Prosocial behavior and altruism: a review of concepts and definitions. Curr Opin Psychol. (2022) 44:124–9. doi: 10.1016/j.copsyc.2021.08.021 34627110

[54] Toi M Batson CD. More evidence that empathy is a source of altruistic motivation. J Pers Soc Psychol. (1982) 43:281–92. doi: 10.1037/0022-3514.43.2.281

